# Health care resource utilization and cost for asthma patients regularly treated with oral corticosteroids – a Swedish observational cohort study (PACEHR)

**DOI:** 10.1186/s12931-018-0855-3

**Published:** 2018-09-03

**Authors:** Christer Janson, Karin Lisspers, Björn Ställberg, Gunnar Johansson, Gunilla Telg, Marcus Thuresson, Helene Nordahl Christensen, Kjell Larsson

**Affiliations:** 1 Department of Medical Sciences, Respiratory, Allergy and Sleep Research, Uppsala University, 751 85 Uppsala, Sweden; 2 Department of Public Health and Caring Sciences, Family Medicine and Preventive Medicine, Uppsala University, Uppsala, Sweden; 3 Statisticon AB, Uppsala, Sweden; 4 AstraZeneca Nordic-Baltic, Södertälje, Sweden; 5 The National Institute of Environmental Medicine, Karolinska Institute, Solna, Sweden

**Keywords:** Severe asthma, Uncontrolled asthma, Health care resource utilization, Oral corticosteroids, Cost

## Abstract

**Background:**

Patients with severe uncontrolled asthma may receive oral corticosteroid (OCS) treatment regularly. The present study investigated the health care resource utilization and cost in regularly OCS treated Swedish asthma patients.

**Methods:**

Primary care medical records data were linked to data from Swedish national health registries. Patients ≥18 years with a drug claim for obstructive pulmonary diseases during 2007–2009 (index date) and a prior asthma diagnosis, were classified by their OCS claims during the 12-months’ post index period: regular OCS equals ≥5 mg per day; periodic OCS less than 5 mg per day; or non-OCS users. Cost of asthma- and OCS-morbidity-related health care resource utilization were calculated.

**Results:**

A total of 15,437 asthma patients (mean age 47.8, female 62.6%), whereof 223 (1.44%) were regular OCS users, 3054 (19.7%) were periodic, and 12,160 (78.7%) were non-OCS users. Regular OCS users were older and more often females, had lower lung function, greater eosinophil count and more co-morbidities at baseline compared with the other groups. Age-adjusted annual total health care cost was three-times greater in the regular OCS group (€5615) compared with the non-OCS users (€1980) and twice as high as in the periodic OCS group (€2948). The major cost driver in the non-OCS and periodic OCS groups were primary care consultations, whereas inpatient costs were the major cost driver in the regular OCS group. The asthma related costs represented 10–12% of the total cost in all three groups.

**Conclusion:**

In this real-life asthma study in Sweden, the total yearly cost of health care resource utilization for a regular OCS user was three times greater than for a patient with no OCS use, indicating substantial economic and health care burden for asthma patients on regular oral steroid treatment.

## Background

Asthma presents with different degrees of severity. The definition of asthma severity has changed from symptom-based to a definition focusing on the intensity of treatment required to achieve good asthma control [1]. The severity range from mild asthma, treated with bronchodilators as needed, to severe asthma treated with high-dosage inhaled corticosteroids (ICS) and additional controller or oral corticosteroids (OCS) [1]. In Sweden, the overall asthma prevalence is approximately 8% [2] and of these, about 4% have severe asthma [3], while studies from other countries have reported a greater severe asthma prevalence of 5–10% out of the asthma population [4]. A study by Bülow et al. yielded that more than 8% of the Danish asthma population had severe asthma, and, of these, almost 40% were uncontrolled [5]. Kerkhof and colleagues recently reported that patients with severe uncontrolled eosinophilic asthma accounted for substantially greater asthma-related health care recourse utilization and costs compared with the overall asthma population [6]. Furthermore, poor asthma control is associated with greater costs, both direct [7, 8] and indirect costs [9]. The estimated cost of asthma in Sweden have been reported to be about SEK 7 billion, a cost that increased considerably with increased disease severity [10].

Patients with severe uncontrolled asthma are more likely to regularly need treatment with OCS compared with patients with mild or moderate disease [11–13], the reason for treatment could be asthma exacerbation or to obtain asthma control. Regular use of OCS in asthma is however associated with greater risks of systemic corticosteroid-related complications [14]. The short- and long-term detrimental adverse effects includes osteoporosis, diabetes and heart failure [15–17] and the consequences appear to be related to the exposure time and OCS dosage [18].

A group of patients that more often are OCS-dependent have high blood eosinophil counts [19]. Increased eosinophils are associated with increased disease severity, more exacerbations and less well-controlled asthma [20–23]. Therefore, these patients may be eligible for treatment with biologics. To our knowledge, recent Swedish data on health care resource utilization and cost in an asthma population on OCS treatment is lacking. The aim of the present study was therefore to investigate the health care resource utilization and costs in real-world Swedish asthma patients regularly treated with OCS.

## Methods

### Study design

In this observational cohort study, primary care medical records data for asthma patients from 36 primary care centers were extracted using an established software system (Pygargus Customized eXtraction0, Program, CXP™) [24], and linked to data from mandatory Swedish national health registries. Centers were selected to cover a representative sample of the Swedish asthma population, by a mix of rural and urban areas, public and private providers and center size. Prior to processing of data, the personal identification numbers were replaced with a study identification number. Data on morbidity were collected from the National Patient Register, containing information from inpatient hospital care (admission and discharge dates, main and secondary diagnoses), and outpatient hospital care (number of contacts and diagnoses as specified by International Classification of Diseases, 10th revision, Clinical Modification (ICD-10-CM codes)). Data on drug claims were collected from the Swedish Prescribed Drug Register (collection date and drug type). The data collection method has been previously described in detail [25]. The Swedish National Board of Health and Welfare performed data linkage and the linked database was managed by the Department of Medical Sciences, Respiratory Medicine at Uppsala University, Sweden. The study protocol was reviewed and approved by the regional ethics committee in Uppsala, Sweden (reference number 2014/446).

### Study population

The study population included males and females ≥18 years of age, who had a record of a drug claim for obstructive pulmonary diseases (Anatomical Therapeutic Chemical (ATC) code R03) during 2007–2009, and a physician-diagnosed asthma (ICD-10 code J45-J46) established prior to drug collection. Patients with a diagnosis of polymyalgia rheumatica (ICD-10 code M35.3) or rheumatoid arthritis (ICD-10 code M05) were excluded. Index date was defined as date of first collection of an R03 drug during 2007–2009 (inclusion period) and a 12-month period after index date was defined as the baseline period to describe the patient characteristics for lung function, comorbidity, and medication and OCS exposure. Patients were followed post baseline (index date+ 365 days) until emigration, death or end of follow up (31st of December 2013).

### Study variables

Patients were classified by their oral glucocorticoids (ATC code: H02AB) pharmacy claims during the 12-months baseline period:

- Regular OCS = OCS claims equivalent to ≥5 mg per day (365 days)
- Periodic OCS = OCS claims equivalent to less than 5 mg per day
- No OCS = no OCS claim

Asthma medications [ATC code] were defined as: inhaled corticosteroids (ICS) [R03BA], short-acting β_2_-agonists (SABA) [R3AC02–03], long-acting β_2_-agonists (LABA) [R03AC12–13,17–18, R03CC12], long-acting muscarinic antagonist (LAMA) [R03BB04], fixed ICS/LABA combination [R03AK], leukotriene receptor antagonists (LTRA) [R03DC]. All other concomitant medications were identified by their respective ATC codes.

Lung function data were assessed from the electronic medical records, and if more than one lung function measurement, expressed as FEV_1_% predicted value, was available during the 12-month post index period, the highest value was used.

Comorbidities (ICD-10 codes) were defined as rhinitis (J30-J32), nasal polyps (J33), acute lower respiratory infections (J20-J22), COPD (J43-J44), pneumonia (J11-J18), diabetes type 2 (E11), metabolic disorders (E70-E90), hypertensive diseases (I10-I15), ischaemic heart disease (I20-I25), heart failure (I50), anxiety and depression (F32), and osteoporosis (M80-M82).

The total, asthma-related, and OCS-morbidity-related (i.e osteoporosis, ischemic heart disease, hypertension, heart failure, diabetes and depression) health care resource utilization (primary care consultations, outpatient specialist care visits to physician, or hospital admissions) were defined by a recorded diagnosis in the medical record or the patient registry. Primary care consultations included all contacts with primary care, including visits and phone calls with doctors, nurses and other health care professionals such as physiotherapist and dietician. Asthma related health care costs were defined as visits with a recorded asthma diagnosis. Asthma related medication cost were defined as ICD-10 code R03.

Costs for hospital admissions and outpatient contacts (including emergency room visits) were estimated using Diagnosis-related groups (DRGs) (DRG weight unit cost for 2014). Drug acquisition costs of all outpatient prescribed drugs were calculated based on the Swedish pharmacy retail prices. Primary care unit costs were based on an average of seven different regional pricelists in Sweden [26]. Swedish krona was converted to Euros using 2017 exchange rate.

### Statistical analyses

Baseline characteristics was described as mean (SD) for continuous variables and absolute and relative frequencies for categorical variables. Comparisons between the three groups at baseline were performed using one-way ANOVA for continuous variables and logistic regression models for categorical variables. Data was presented as crude and adjusted for age. Because of differences in age distribution between groups, the health care recourse utilization and cost were age-standardized using a direct age standardization (i e all observations were weighted relative to the proportion of patient in the specific age group in the total population). Statistical analyses were performed using SAS version 9.3 and R version 3.2.3.

## Results

This study included a total of 15,437 asthma patients (mean age 47.8, female 62.6%), whereof 223 (1.44%) patients were on regular OCS treatment at baseline, 3054 (19.7%) were periodic OCS users and 12,160 (78.7%) were non-OCS users (Table 1). Median follow-up time was 5.65 years, 5.75 for the regular OCS group, 5.71 for the periodic group and 5.63 for the non-OCS users. Mean age was significantly greater in the regular OCS group compared to the periodic and non-OCS groups, 62.3 vs 49.4 and 47.2 years respectively. Female sex was more common in the periodic OCS (66%) compared to the non-OCS (62%) and the regular OCS (57%) groups. Lung function was lower in the regular OCS group compared to both the periodic and the non-OCS groups, while eosinophil counts were greater in the regular OCS group compared to the two other groups (Table 1). Inhaled corticosteroids (ICS) were used by 82% of the non-OCS users, 88% of the periodic OCS group and 90% of the patients in the regular OCS group. ICS plus long-acting beta-agonists (LABA), in fixed combination or as mono therapy, were more commonly used in the regular OCS group (72%) compared to the periodic OCS (60%) and the non-OCS users (45%) (Table 1). There was a significant difference between the groups in the percentage of patients with co-morbidities at baseline, with more frequent COPD, pneumonia, diabetes, hypertensive disease, ischaemic heart disease, heart failure, cerebrovascular disease, depression and osteoporosis in the regular OCS group compared with the two other groups (Table 2).

**Table 1:** Baseline characteristics

	Regular OCS use *n* = 223	Periodic OCS use *n* = 3054	No OCS use *n* = 12,160	*P*-value
Age, mean (SD)	62.3 (15.9)	49.4 (18.2)	47.2 (19.0)	< 0.001
Female, *n* (%)	127 (57.0)	2028 (66.4)	7508 (61.7)	< 0.001
BMI, mean (SD), *n*	28.1 (6.0), 146	27.5 (6.0), 1629	27.3 (5.6), 6432	0.081
FEV1% predicted^a^, mean (SD), *n*	66.9 (20.9), 35	79.1 (23.8), 365	87.4 (20.2), 940	< 0.001
FVC % predicted^a^, mean (SD), *n*	85.1 (21.9), 26	90.2 (19.8), 294	95.2 (16.9), 817	< 0.001
FEV1/FVC, mean, (SD), *n*	0.67 (0.14), 27	0.72 (0.16), 307	0.78 (0.13), 833	< 0.001
Neutrophils, cells/mm3, mean (SD), *n*	6.60 (3.02), 79	5.78 (3.06), 481	5.09 (2.54), 973	< 0.001
Eosinophils, ×10^3^ cells/μL, mean (SD), *n*	0.49 (0.96), 95	0.35 (0.53), 665	0.30 (0.41), 1798	< 0.001
ICS, *n* (%)	201 (90.1)	2680 (87.8)	9939 (81.7)	< 0.001
LABA, *n* (%)	168 (75.3)	1881 (61.6)	5701 (46.9)	< 0.001
ICS + LABA, fixed or mono, *n* (%)	160 (71.7)	1833 (60.0)	5442 (44.8)	< 0.001
Short-acting ß2-agonists, *n* (%)	152 (68.2)	2172 (71.1)	7742 (63.7)	< 0.001
Leukotriene receptor antagonists, *n* (%)	41 (18.4)	425 (13.9)	574 (4.7)	< 0.001
Long-acting muscarinic antagonist, *n* (%)	29 (13.0)	214 (7.0)	374 (3.1)	< 0.001
Anti-IgE treatment, *n* (%)	1 (0.4)	3 (0.1)	1 (0.0)	0.010
Bisphosphonates, *n* (%)	44 (19.7)	75 (2.5)	160 (1.3)	< 0.001
Betablockers, (%)	65 (29.1)	412 (13.5)	1418 (11.7)	< 0.001

^a^Post broncodilator

*Fixed* = Fixed combination inhalers

*Mono* = Mono therapy inhalers

**Table 2:** Baseline comorbidities

	Regular OCS use *n* = 223	Periodic OCS use *n* = 3054	No OCS use *n* = 12,160	*P*-value
Rhinitis, *n* (%)	34 (15.2)	660 (21.6)	2181 (17.9)	< 0.001
Nasal polyps, *n* (%)	11 (4.9)	147 (4.8)	231 (1.9)	< 0.001
Other acute lower respiratory infections, *n* (%)	60 (26.9)	938 (30.7)	2633 (21.7)	< 0.001
COPD, *n* (%)	62 (27.8)	423 (13.9)	999 (8.2)	< 0.001
Pneumonia, *n* (%)	47 (21.1)	527 (17.3)	1426 (11.7)	< 0.001
Diabetes type 2, *n* (%)	26 (11.7)	170 (5.6)	662 (5.4)	0.002
Hypertensive diseases, n (%)	88 (39.5)	696 (22.8)	2596 (21.3)	< 0.001
Ischaemic heart disease, *n* (%)	43 (19.3)	237 (7.8)	793 (6.5)	< 0.001
Heart failure, *n* (%)	35 (15.7)	160 (5.2)	425 (3.5)	< 0.001
Malignant neoplasm, *n* (%)	2 (0.9)	1 (0.0)	7 (0.1)	0.023
Cerebrovascular diseases, *n* (%)	17 (7.6)	70 (2.3)	297 (2.4)	< 0.001
Depression, *n* (%)	39 (17.5)	449 (14.7)	1623 (13.3)	0.042
Osteoporosis, *n* (%)	23 (10.3)	72 (2.4)	218 (1.8)	< 0.001

**Fig. 1:**
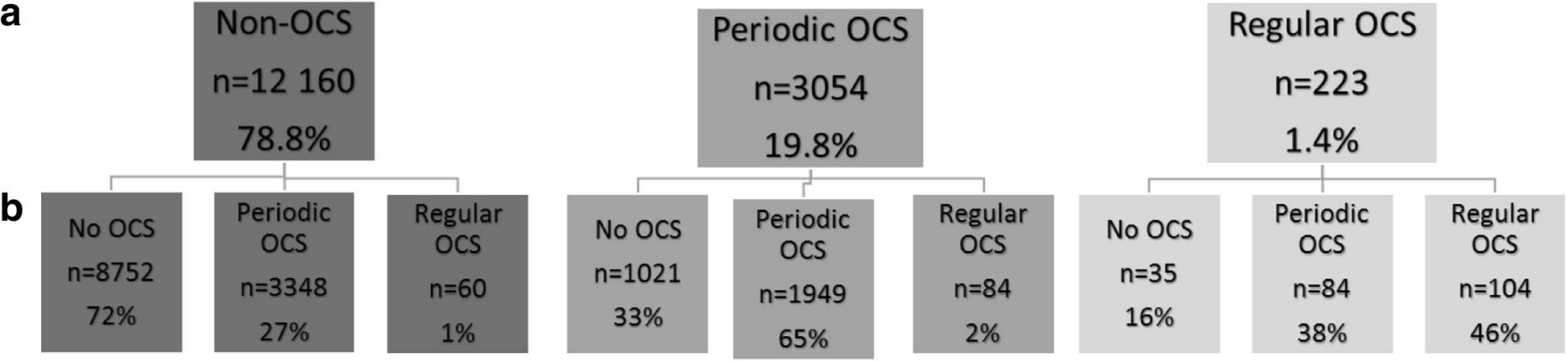
Asthma patients (number and percentage) **a** classified by OCS use during the 12-months baseline period, and **b** by their mean annual OCS use during follow-up

During follow-up, 46% of the regular OCS patients continued on regular OCS and 38% switched to periodic OCS use. Sixteen percent of the regular OCS patients did not claim any OCS at all during the follow-up (Fig. 1). In the non-OCS group, 72% did not claim any OCS during follow up, while periodic OCS use was recorded in 27% and regular use in 1% of the patients. In the periodic OCS group, 33% did not claim an OCS during follow up while 65% continued to be periodic OCS users and 2% became regular-OCS users (Fig. 1).

**Fig. 2:**
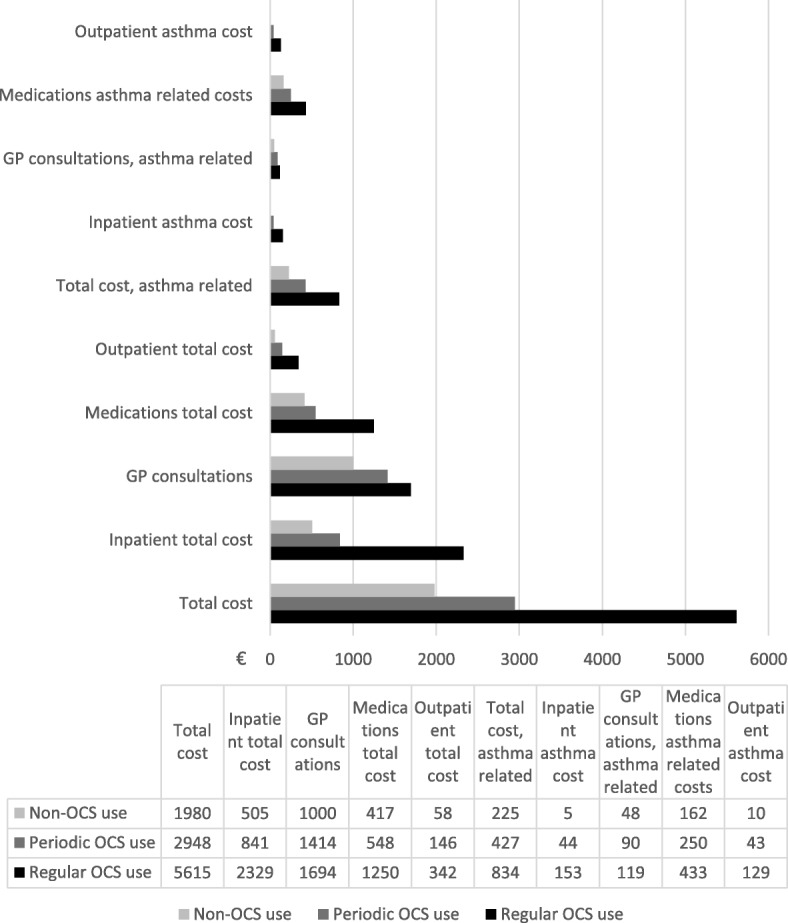
Mean yearly cost (Euro) during follow-up for the Non-OCS, Periodic OCS and Regular OCS groups. Data weighted according to age group

Age adjusted mean total health care cost was twice as great per year in the regular OCS group (€5615) compared with the periodic OCS group (€2948) and three times as great as for the non-OCS group (€1980) during follow-up. The major cost driver in the non-OCS and periodic OCS groups were primary care consultations, €1000 and €1414 respectively, whereas inpatient costs were the major cost driver in the regular OCS group with €2329. The asthma related costs were 10–12% of the total cost in all three groups (Fig. 2). In-patient annual asthma related costs were four times greater in the regular OCS group (€153) compared to the periodic OCS group, and even more pronounced compared to the non-OCS users (Fig. 2).

**Fig. 3:**
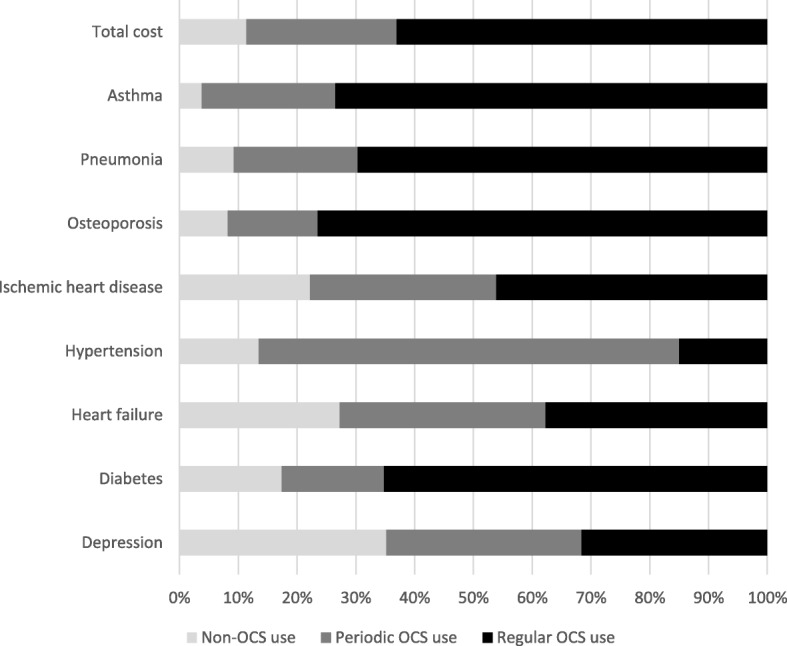
Age adjusted percentage of costs of asthma and OCS-associated comorbidities per 100 patient years, by Non-OCS, Periodic OCS and Regular OCS groups

In addition, age adjusted total annual cost of comorbidities associated with OCS use were three times greater in the regular OCS group compared to the non-OCS users and more than double as great as in the periodic OCS group during follow-up. The regular OCS group accounted for almost 80% of the total yearly event cost of pneumonia, and > 80% of the total outpatient osteoporosis costs (Fig. 3). When excluding patients with a concomitant diagnosis of COPD (*n* = 62), the age adjusted annual total health care cost remained greater in those on regular OCS treatment compared to those in the periodic OCS and non-OCS groups (€4280 vs. €2401 and €1747, respectively).

## Discussion

The present observational cohort study in Swedish primary care included more than 15,000 asthma patients across the asthma severity continuum. Of these, 223 patients (1.44%) were on regular treatment with OCS during 12 months post index. Regular OCS users were older, had lower lung function, higher eosinophil count and more comorbid disease compared with the non-OCS users. Almost half of the regular OCS users continued on regular OCS throughout the five-year observation period and two out of five continued as periodic OCS users. Age adjusted annual health care costs for the regular OCS group was three times greater compared with the non-OCS user, where the majority of the costs were not directly asthma related.

The mean annual cost for the regular OCS group was €5615, which was three times greater than for the non-OCS group, supporting already available data on the increased health care cost associated with OCS use [27] and poorly controlled asthma [7]. Indirect costs could not be captured in our study. Total drug costs accounted for one fifth and inpatient cost accounted for one third of the total cost, whereof only 12% were related to asthma. In a study by Accordini et al. it was shown that drug costs accounted for 16% of the total cost, which is similar to our findings, whereas hospitalization costs were much lower compared to our results. This study did, however, include indirect costs, accounting for half of the mean total cost [8].

In a previous study in Swedish primary care, we have reported a severe asthma (GINA step 4 and 5) [1] prevalence of 4%, whereof more than half had uncontrolled asthma [3]. Application of these results to the present study population, for which patients were included based on OCS claims without controlling for previous ICS dosage or asthma control, an estimated one-third of the severe uncontrolled asthma patients would be long-term regular OCS users. This is well in keeping with what has been reported by others with OCS being used in between 30 and 40% of the severe uncontrolled asthma patients [11, 12].

Uncontrolled asthma is associated with a reduction in Quality of Life and activity functions, including limitations in daily activities, and activity avoidance [28]. Use of OCS has previously been associated with greatly enhanced risk of osteoporosis and diabetes [15, 16] and in our study, the majority of costs for these diseases were attributed to the regular OCS group. However, the cost of both depression and heart failure seemed to be evenly distributed between the three groups in the present study, contradicting previous reports [17, 29]. Nasal polyps were significantly more frequent in the periodic and regular OCS groups compared with the non-OCS group, indicating a more eosinophilic phenotype.

The present study investigated total annual OCS exposure, as the detrimental effect of OCS have been demonstrated to have been dose-dependent [15, 18]. Thus, the reason for OCS treatment was not taken into account when classifying patients into the three OCS groups, and treatment could have been initiated as courses of OCS due to asthma exacerbations or as longer-term maintenance treatment. An OCS dosage of 5 mg per day would amount to an annual OCS dosage of 1825 mg, and patients with corresponding claims were classified as regular OCS users. Previous studies have reported that patients with severe uncontrolled asthma may require several oral steroid bursts yearly [30, 31]. These patients could potentially be exposed to an annual OCS dosage of the same magnitude as patients with longer term low dosage maintenance treatment. When excluding patients with a concomitant COPD diagnosis the relative difference in cost between the groups remained, thereby indicating that the observed difference was not driven by patients with a concomitant COPD diagnosis.

In the present study, the regular OCS users had greater baseline blood eosinophil counts compared with the periodic and non-OCS users. The high health care cost associated with regular OCS use observed in our study, in combination with the known adverse effects of OCS, emphasizes the importance of steroid sparing strategies in the treatment of patients with severe asthma. New biologic treatments, targeting severe uncontrolled eosinophilic asthma have recently become available, and data show that in addition to effectively decrease exacerbations, these treatments also result in reduced OCS need [32, 33]. At present, to be eligible for treatment with these compounds, patients should fulfil criteria’s such as specific eosinophil counts and previous exacerbations. It has, however, been suggested that a target population for biologic treatments may be asthma patients with regular OCS use [34].

This is an observational cohort study with linkage of data from electronic medical record and national health registries, a design that comes with several limitations. A key limitation is that medication use is based on prescription claims, which do not fully reflect how patients actually use medications. Data retrieval is limited to the variables recorded in the databases and personal and phenotypic characteristics were available for a limited number of patients only. The Swedish Prescribed Drug Register only includes drugs claimed at the pharmacy directly by the patients, and thus does not cover use of biologics as these are administered at a hospital. The number of potential users of biologics in this study would, however, likely be very low as the patients in this study were identified in primary care. Another key limitation of this study is the disparity between the regular and the non-OCS users at baseline. Comorbidities were more frequent the regular OCS group which also was older, why age adjusted analyses were included. Another important limitation is that it cannot be ruled out that some use of OCS was indicated by other diseases than asthma. To mitigate for this potential bias, patients with rheumatoid arthritis and polymyalgia rheumatica were excluded.

This study also has several important strengths, not the least the lack of selection bias and the linkage of electronic primary healthcare data to mandatory health registers with high coverage and quality. It can therefore be expected that the generalizability of our findings in the management of asthma patients in a primary care setting is high.

## Conclusion

The total yearly cost of health care resource utilization for a Swedish asthma patient on regular OCS treatment was three times greater than for a patient with no OCS use, indicating substantial economic and health care burden for asthma patients on regular oral steroid treatment.

## Abbreviations

ATC: Anatomical Therapeutic Classification System code
CI: Confidence interval
COPD: Chronic obstructive pulmonary disease
FEV: Forced expiratory flow
FVC: Forced vital capacity
ICD-10-CM: International Classification of Diseases 10 Clinical Modification
ICS: Inhaled steroid
LABA: Long-acting beta-agonists
LTRA: Leukotriene modifiers
RR: Relative risk
SABA: Short-acting beta-agonists
SD: Standard deviation
